# Prognostic role of long non-coding RNA USP30-AS1 in ovarian cancer: insights into immune cell infiltration in the tumor microenvironment

**DOI:** 10.18632/aging.205262

**Published:** 2023-12-04

**Authors:** Jian Xiong, Junyan Chen, Xiang Sun, Rui Zhao, Kefei Gao

**Affiliations:** 1 Department of Obstetrics and Gynecology, Guangzhou Women and Children’s Medical Center, Guangzhou Medical University, Guangzhou 510623, China; 2 China Medical University, Shenyang 110122, China

**Keywords:** ovarian cancer, tumor microenvironment, USP30-AS1, immune cell infiltration, LncRNA

## Abstract

Ovarian cancer represents a formidable gynecologic malignancy bearing a dismal prognosis owing to the dearth of reliable early detection approaches and a high recurrence rate. Long non-coding RNAs (lncRNAs) have garnered immense attention as key orchestrators involved in diverse biological processes and take part in cancer initiation and progression. The present study investigated the potential significance of lncRNA USP30-AS1 in ovarian cancer prognosis, as well as its putative association with immune cell infiltration in tumor immune microenvironment (TIME). By analyzing publicly available datasets, we identified six lncRNAs with prognostic prediction ability, including USP30-AS1. The results revealed a significant positive correlation of USP30-AS1 expression with the infiltration of immune cells such as Th1 cells, TFH, CD8 T cells, B cells, antigen-presenting dendritic cells (aDC), and plasmacytoid dendritic cells (pDC) in ovarian cancer specimens. These findings provide compelling evidence of the potential involvement of lncRNA in the regulation of the TME in ovarian carcinoma. The outcomes from this study underscore the potential of USP30-AS1 as a promising prognostic biomarker for ovarian cancer. Additionally, the findings offer significant insights into the plausible role of lncRNAs in modulating immune activities, thus adding to our understanding of the disease biology. Additional investigations are necessary to unravel the molecular mechanisms underpinning these connections and validate the results seen in independent cohorts and experimental models.

## INTRODUCTION

Ovarian carcinoma (OC) is one of the most lethal gynecological malignancies and a paramount public health concern worldwide [1–3]. Ovarian carcinoma is often diagnosed at advanced stages due to its asymptomatic nature and the absence of effective early detection methods. Despite recent advancements in surgical and chemotherapeutic treatments, the overall survival (OS) rate of ovarian cancer patients remains poor, with only 46% surviving beyond 5 years [4, 5]. The high mortality rate associated with ovarian cancer highlights the urgent need for innovative diagnostic and treatment strategies.

The role of the tumor immune microenvironment (TIME) in shaping cancer progression and determining the efficacy of cancer treatment is becoming increasingly recognized [6, 7]. The tumor microenvironment (TME) consists of a multifaceted network of immune cells, stromal cells, extracellular matrix constituents, and signaling molecules collaboratively control the development, advancement, and metastasis of the tumor [8–10]. Recent studies have highlighted the significance of immune cell infiltration within the TME, as it has been demonstrated to impact cancer prognosis and treatment effectiveness [11–13]. For example, an association has been demonstrated between a more favorable prognosis and the presence of cytotoxic T lymphocytes and so on. The converse has been observed with the presence of immunosuppressive cells such as Tregs, with poorer patient outcomes linked to the latter.

Long non-coding RNAs (lncRNAs) are a group of RNA molecules that, unlike messenger RNAs (mRNAs), do not encode proteins but are critical in different biological processes, including cellular proliferation and growth, cellular differentiation, programmed cell death, and immunological response regulation. The aberrant expression of lncRNAs has been implicated in the progression of numerous cancers, such as ovarian cancer [14, 15]. Notably, lncRNAs have been proven to regulate the infiltration of immune cells in TIME, and influence both cancer prognosis and therapeutic responses [16, 17].

One such lncRNA, USP30-AS1, has been reported to be associated with Th1 cells and shows strong prognostic prediction ability in ovarian cancer [18, 19]. However, the underlying molecular mechanisms through which USP30-AS1 influences immune cell infiltration and ovarian cancer prognosis remain largely unknown. This investigation primarily investigated the potential implications of USP30-AS1 in ovarian cancer prognosis and its putative effects on immune cell infiltration. USP30-AS1 has emerged as a promising lncRNA molecule with an important role in the pathogenesis of various cancers, including ovarian cancer. Our research aimed to explore the underlying association between USP30-AS1 and immune activities in ovarian carcinoma. We examined a variety of immune cells, such as Th1 cells and CD8 T cells, which play crucial roles in regulating TIME and tumor progression. Our study not only aims to enrich our understanding of the molecular mechanisms underpinning ovarian cancer but also hopes to identify potential novel therapeutic targets for this cancer. This study provides valuable insights into how USP30-AS1 influences immune cell infiltration and aims to provide a comprehensive understanding of its role in tumor progression and its potential application in targeted therapies for ovarian cancer.

Computational biology and high-throughput sequencing techniques have currently become integral and have revolutionized biomedical research methodologies, enabling us to process large amounts of genetic and genomic data with high precision and efficiency [20–22]. To identify biomarkers in diverse tumors, Wang et al. employed computational biology approach, such as weighted correlation network analysis (WGCNA), hence laying a secure, scientific groundwork for investigating the causes of disease progression [23, 24]. Consequently, the goal of this scientific study was to provide novel insights into the complex mechanisms underlying the involvement of USP30-AS1 in the prognosis of ovarian cancer and its influence on the TIME. The findings may aid in expanding the current knowledge base and ultimately enhance the development of innovative treatments for this cancer. Elucidating the potential associations between USP30-AS1 expression and clinical outcomes would facilitate the emergence of pioneering diagnostic and therapeutic approaches that have the potential to enhance the clinical outcomes and survival rates of patients with ovarian cancer.

## MATERIALS AND METHODS

### Data acquisition and preprocessing

We retrieved processed whole transcriptome data for 34 cancer cohorts, including both normal and tumor tissues, from The Cancer Genome Atlas (TCGA) database through the UCSC XENA website (https://xena.ucsc.edu/). Clinical data and gene mutation data for Ovarian Cancer (TCGA-OV) were also obtained from the UCSC XENA platform. In addition, transcriptome data for ovarian cancer were sourced from the Genotype-Tissue Expression (GTEx) database (https://www.gtexportal.org/home/index.html). These databases are open-access, allowing unrestricted access and analysis, thus obviating the need for ethical approval. We ensured that data retrieval and analysis procedures adhered to the ethical requirements of the respective databases and journals.

Transcriptomic data for tumor and normal tissue specimens were acquired from gene expression data records, following TCGA's unique specimen naming conventions. Gene expression data was standardized using Log_2_(FPKM+1). Information on immune therapy cohorts was obtained from the “IMvigor210” dataset. The list of genes related to TLS (Tertiary Lymphoid Structures) was derived from literature searches, and the detailed gene list is provided in Supplementary Table 1.

### Differential TLS gene selection and further analysis

Initially, we integrated ovarian cancer whole transcriptome data from GTEx and TCGA (Normal=88, Tumor=379) and employed the “limma” package to analyze the differential expression of TLS genes between tumor and normal groups. The selection criteria were set at false discovery rate (FDR) < 0.05 and | logFC | > 1. A volcano plot was generated to visually represent the differentially expressed genes. Subsequently, we utilized the protein-protein interaction visualization feature of the STRING website (https://www.string-db.org/) to illustrate the interaction relationships among differential TLS genes. Univariate Cox analysis was employed to assess the impact of differential TLS genes on prognosis, considering genes with p < 0.01 as those significantly affecting prognosis. Subsequently, a Venn diagram was employed to identify the intersection between differential TLS genes and prognostically impactful TLS genes. The resulting genes were then visualized in a heatmap to observe their expression patterns in tumor and normal tissues. Based on the median expression values of the identified genes, tumor patients were stratified into high and low expression groups. Kaplan-Meier (KM) curves were applied to analyze the overall survival differences between the two expression groups for each gene. Finally, leveraging IMvigor immune therapy cohort data, we analyzed the expression differences of each gene between effective and ineffective immune therapy groups, presenting the results through violin plots.

### Non-negative matrix factorization (NMF) analysis and subsequent investigations

Building upon the TLS genes identified through the intersection of the Venn diagram in Section 2.2, we conducted NMF clustering analysis on ovarian cancer patients from TCGA. The final number of clusters was determined by selecting the value of 'k' that exhibited a “high cohesion, low coupling” pattern in the consensus matrix plot. Subsequently, Kaplan-Meier survival curves were generated for the two clusters. Following this, we conducted a comprehensive analysis of the tumor immune microenvironment (TIME) for these two clusters, encompassing the evaluation of Immune Score, Stromal Score, Estimate Score, and tumor purity. We further assessed the expression profiles of eight immune molecules (IgG, HCK, MHC-I I, LCK, STAT1, Interferon, B7-CD28, TNF) in these two clusters, visualizing differences in survival status, cancer stage, TIME, and the expression of these eight immune molecules using heatmaps. Single-sample Gene Set Enrichment Analysis (ssGSEA) was employed to investigate differences in the infiltration of 29 immune cell types between the two clusters. Additionally, a differential gene expression analysis was conducted on these two clusters, with the selection criteria set at | logFC | > 1 and P-adj < 0.05. The resultant differentially expressed genes were subjected to Kyoto Encyclopedia of Genes and Genomes (KEGG) and Gene Ontology (GO) pathway enrichment analyses [25–27].

### Construction and validation of a prognostic model based on TLS gene-associated lncRNA

Utilizing the transcriptome data of TCGA-OV tumor patients, we conducted an analysis of lncRNAs co-expressed with TLS genes, as identified through the intersection of the Venn diagram in Section 2.2. LncRNAs were selected based on the criterion of a correlation coefficient greater than 0.35 and a p-value less than 0.05 for each gene. Subsequently, Spearman correlation analysis was performed for each TLS gene with its four strongest correlated lncRNAs, and the results were visualized using scatterplots. Next, the identified lncRNAs were incorporated into The Least Absolute Shrinkage and Selection Operator (LASSO) machine learning algorithm. Ten-fold cross-validation was employed for further refinement of the selected lncRNAs, ultimately leading to the construction of a risk model. Our random seed for this analysis was set to “1997.” The risk score was defined by multiplying the expression value of each gene (i) by its corresponding coefficient (βi), as follows:

$$\begin{array}{c} \text{Risk}\;\text{score}=\\ \sum_{\text{i}=1}^{\text{n}}\left[expression\;value\;of\;gene_{i}*\beta_{i}\right] \end{array}$$ (1)

Where β_i represents the coefficient corresponding to the model gene [28–30]. Subsequently, we performed univariate Cox analysis on the model lncRNAs to assess their impact on prognosis and conducted Kaplan-Meier survival curve analysis to evaluate the influence of each lncRNA's expression on prognosis. Receiver operating characteristic curve (ROC) analysis was employed to assess the model's performance in predicting 2-year, 3-year, and 5-year outcomes. An area under the curve (AUC) greater than 0.6 indicated good predictive performance. Patients were then categorized into high-risk and low-risk groups based on the median risk score, and Kaplan-Meier analysis was performed separately for overall survival (OS) and progression-free survival (PFS) in these two groups. Furthermore, a risk factor plot was generated to illustrate how patient survival time, survival status, and the expression of model lncRNAs change with increasing risk scores. Additionally, we utilized Cytoscape software to visualize the expression correlations between TLS genes and model lncRNAs, which were presented using chord diagrams to enhance interpretability and understanding.

### Establishment and validation of the prognostic nomogram

We integrated age, stage, grade, and risk score to comprehensively construct a nomogram model for predicting patient survival at 2, 3, and 5 years. To assess the accuracy of the model, we plotted calibration curves at 2, 3, and 5 years to observe the extent of deviation from the ideal values. To validate the clinical utility of the model, we performed a 5-year Decision Curve Analysis (DCA) for age, stage, grade, risk score, and the nomogram. Finally, we conducted three-dimensional Principal Component Analysis (PCA) separately for the entire transcriptome genes, TLS genes, TLS-related lncRNA genes, and model lncRNA genes. This allowed us to examine whether there were significant differences in the three-dimensional data space dimensions between the high-risk and low-risk sample groups.

### Multiple analytical approaches reveal the intrinsic mechanisms of TLS-related lncRNAs

In our study, we conducted an immune subtype analysis on the TCGA-OV dataset, eliminating subtypes with limited sample sizes. We then proceeded to analyze the differences in lncRNA model risk scores among the remaining subtypes. Utilizing Sankey diagrams, we illustrated the associations between NMF subtypes, risk score risk groups, and immune subtypes. Subsequently, we performed differential analysis of risk scores between groups categorized by treatment outcomes and tumor recurrence status. We employed the “oncopredict” tool to investigate the sensitivity differences of 200 drugs between the two risk groups. Violin plots were created to visualize the sensitivity differences of three specific drugs: LCL161, Ribociclib, and Topotecan. We conducted differential analysis between the two risk groups and subjected the obtained genes to Gene Ontology (GO) analysis. The analyses were carried out using packages such as “limma”, “scales”, “ggplot2”, “ggtext”, “reshape2”, “tidyverse”, and “ggpubr”.

Furthermore, we conducted differential analysis of 16 immune cell infiltrations between the two risk groups and visualized the results using box plots. Subsequently, we employed Weighted Correlation Network Analysis (WGCNA) to identify lncRNAs co-expressed with the ssGESA scores of various immune cells. The relevant parameters included a cutline of 600 and a power value of 4. We selected the immune cell with the strongest statistical significance from the previous two risk group analyses as the target phenotype for further analysis. We identified the WGCNA module with the strongest correlation to this immune cell phenotype and intersected the lncRNAs within this module with the model lncRNAs. We conducted an analysis of immune checkpoints based on the obtained lncRNAs. Additionally, we performed differential expression analysis of the obtained lncRNAs between different risk groups, followed by Kaplan-Meier survival curve analysis for progression-free survival (PFS) and TIDE analysis for Exclusion scores in different risk groups. Based on these three analyses, we filtered out lncRNAs without statistical significance and compared the remaining lncRNAs for their expression differences between different grades and between tumor and normal tissues. We selected USP30-AS1 for further analysis.

To stratify OC patients into two groups based on the median expression value of USP30-AS1, we analyzed the most significantly mutated genes between the two groups and visualized them using waterfall plots. Subsequently, we performed multiple Gene Set Enrichment Analyses (GSEA) to observe enriched pathways in these two groups. We selected the most significantly mutated genes from the waterfall plot and retrieved their immunohistochemistry images in normal ovarian tissues and cancer tissues from The Human Protein Atlas (HPA) website (https://www.proteinatlas.org/) [31]. Finally, we generated scatter plots to illustrate the correlation between USP30-AS1 and six immune cell types in the TCGA-OV dataset.

### Pan-cancer analysis of USP30-AS1

To understand the expression pattern of USP30-AS1 in other cancers, we downloaded processed transcriptomic data of 34 cancer cohorts, including both normal and tumor tissues, from the UCSC XENA website (https://xena.ucsc.edu/). Subsequently, we conducted differential analysis of USP30-AS1 expression between tumor and normal tissues in each cancer cohort and visualized the results using violin plots. We employed the “xCELL” algorithm from the R package “IOBR” (version 0.99.9) to calculate scores for 67 immune cell infiltration in each cancer cohort [32]. Next, we computed the correlation between immune cell infiltration scores and USP30-AS1 expression levels in each cancer cohort using the “Pearson” correlation method. A p-value less than 0.05 was considered statistically significant for the correlation between immune cell infiltration scores and USP30-AS1 expression levels. We visualized the correlation between each immune cell score and USP30-AS1 expression using heatmaps for each cancer cohort. Finally, we selected the cancer cohort with the highest absolute correlation coefficient between Th1 cell score and USP30-AS1 expression. We illustrated the correlation between Th1 cell score and USP30-AS1 expression using scatter plots to visualize the relationship.

### Cell culture, total RNA extraction and RT-qPCR

We purchased three human ovarian cancer cell lines, SK-OV-3, OVCAR-3, CAOV-3, and one human normal ovarian epithelial cell line, IOSE-29, from the Cell Bank of the Chinese Academy of Sciences. SK-OV-3 cells were cultured in McCoy's 5A medium (HyClone, USA), OVCAR-3 cells were cultured in Roswell Park Memorial Institute (RPMI-1640) medium (HyClone, USA), CAOV-3 cells were cultured in Dulbecco's Modified Eagle Medium (DMEM, HyClone, USA), and IOSE-29 cells were cultured in a 1:1 mixture of MCDB 105 and M-199 media (HyClone, USA). All media were supplemented with 10% fetal bovine serum (FBS, BI, USA), 100 U/ml penicillin (HyClone, USA), and 100 μg/ml streptomycin (HyClone, USA). All cell lines were maintained in a cell culture incubator at 37° C with 5% CO_2_.

For total RNA extraction, we employed trypsin (BI, USA) to digest the cells grown in cell culture flasks, and collected them in EP tubes. Subsequently, 900 μl of Trizol (Takara, Japan) was added to lyse the cells, followed by a 5-minute incubation on ice. Then, 200 μl of chloroform (SINOPHARM, China) was added, and vigorous shaking was performed, followed by centrifugation at low temperature for 15 minutes. The clear aqueous phase was carefully transferred to a fresh tube, and an equal volume of isopropanol and absolute ethanol (SINOPHARM, China) was added successively. After each addition, the mixture was incubated on ice for 5 minutes, followed by centrifugation at low temperature. The organic solvents were discarded, and the RNA pellet obtained was air-dried, resembling a feather-like appearance. The RNA pellet was dissolved in an appropriate amount of DEPC-treated water, and the concentration was measured. Subsequently, following the manufacturer's instructions, cDNA synthesis was performed using the PrimeScript RT Kit (TaKaRa, Japan). For subsequent real-time fluorescence quantitative PCR, we utilized the SYBR GreenER Supermix (TaKaRa, Japan) kit. The PCR reactions were conducted on a 7500 Real-Time PCR System (Thermo Fisher Scientific, USA). The PCR reaction conditions comprised an initial denaturation step at 95° C for 1 minute, followed by 35 cycles of 95° C for 90 seconds, 60° C for 30 seconds, and 72° C for 30 seconds, and a final extension step at 72° C for 10 minutes. The relative expression of USP30-AS1 was analyzed using the 2^-ΔΔCt^ method, with β-actin as the reference gene for normalization. The primer sequences used were as follows: β-actin: Forward: 5’- CCTGGCACCCAGCACAAT-3’, Reverse: 5’- GGGCCGGACTCGTCATAC-3’; USP30-AS1: Forward: 5rward: 5’- CCTGGCACCCA -3rward: 5rward: TGAAAACCAAGCAGCCCCA -3AA.

### Statistical analysis

For statistical analysis, R (version 4.1.0) was utilized. The two groups were contrasted using the Student’s t-test or Wilcoxon test. Comparisons between several groups were evaluated using the Kruskal-Wallis test. Kaplan-Meier plots, which show survival curves, were used to compare them to the log-rank test. Results with a p-value < 0.05 were considered significant from a statistical perspective.

## RESULTS

### Differential selection and further analysis of TLS genes

**Figure 1:**
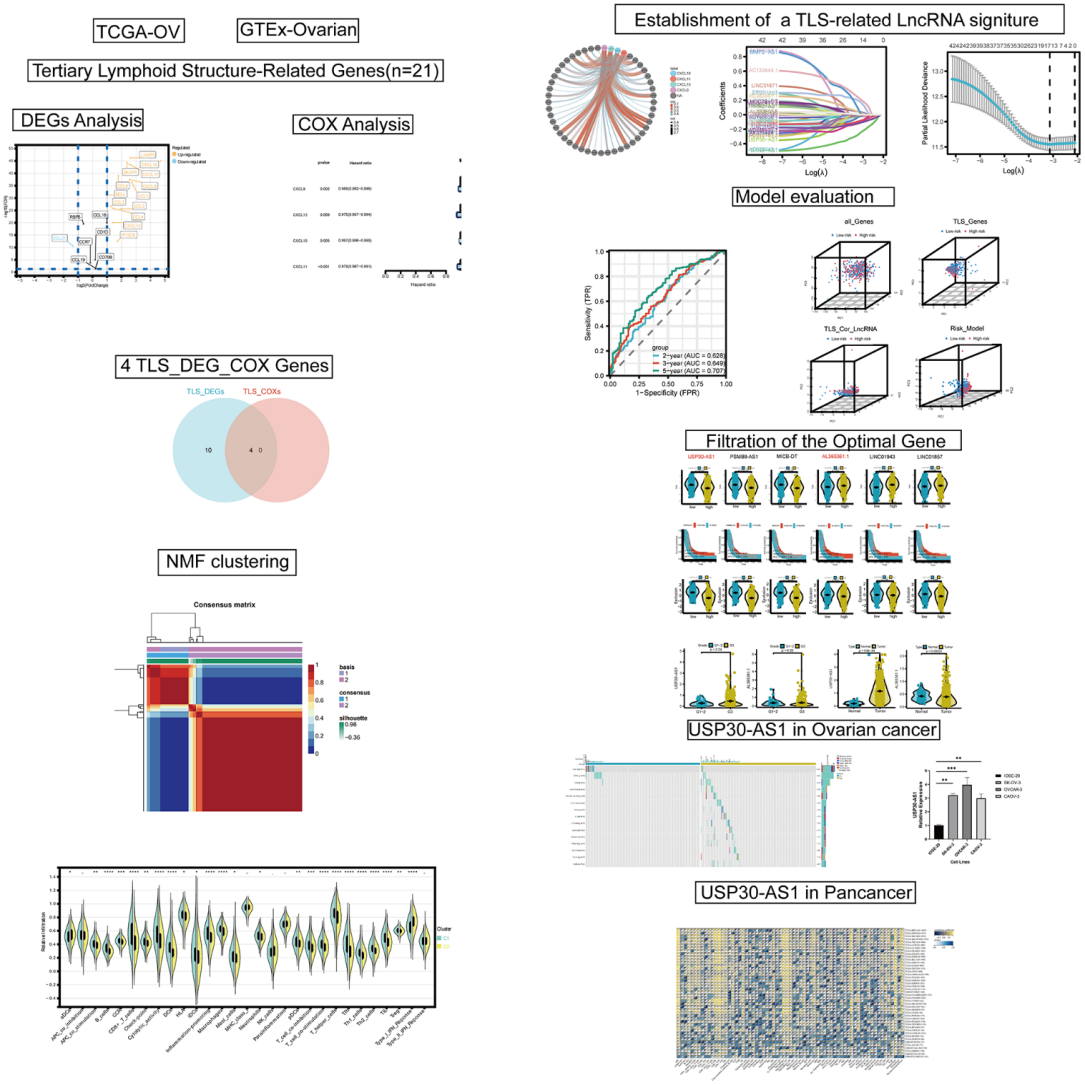
The flow chart of this study.

**Figure 2:**
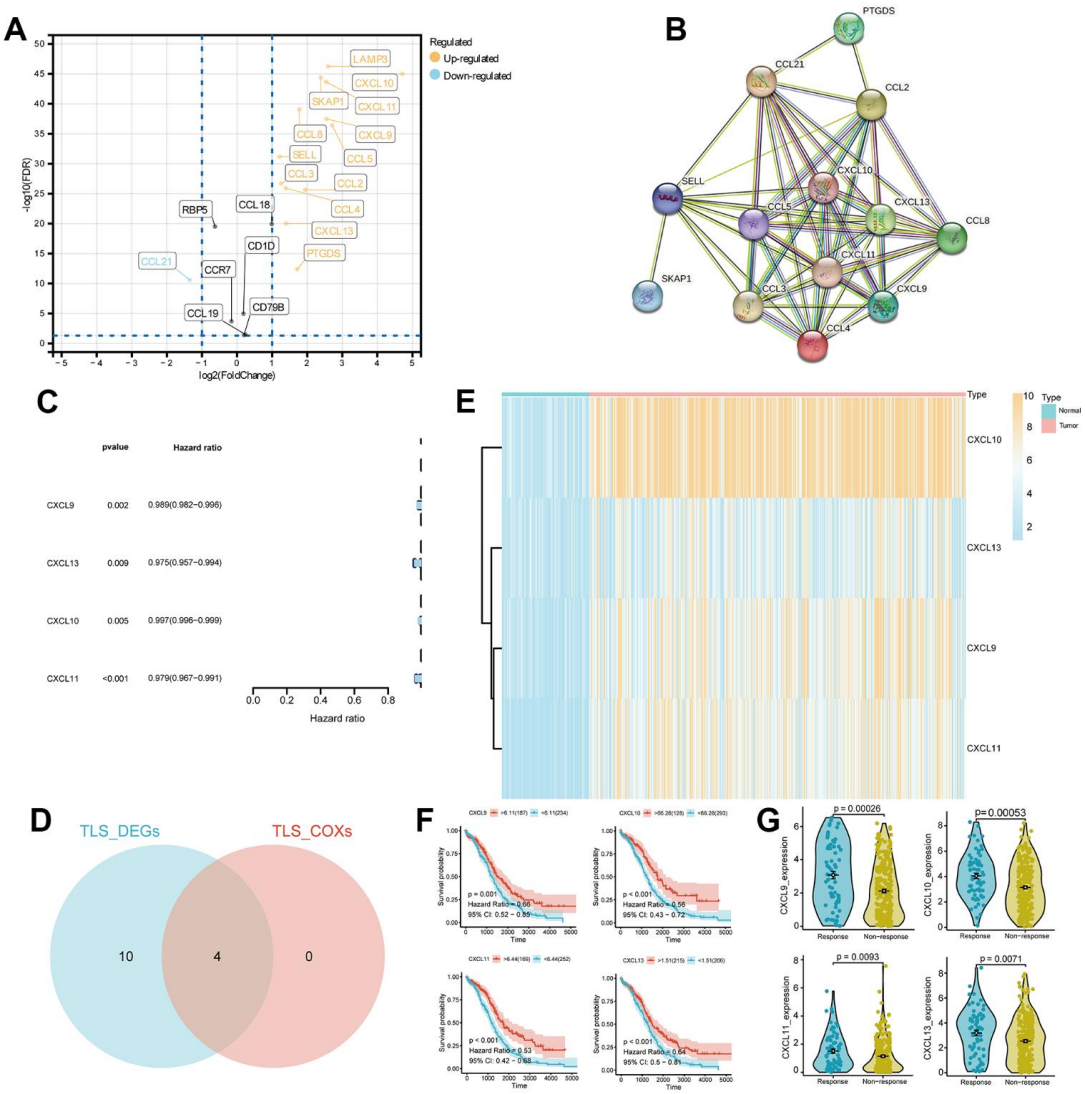
**Differential expression analysis and identification of TLS_differentially expressed genes (DEGs) in ovarian cancer.** (**A**) Volcano plot of DEGs in ovarian cancer with screening criteria fdr < 0.05 and |logFC| > 1. (**B**) Protein-protein interaction (PPI) analysis of the 14 DEGs. (**C**) Univariate COX analysis of the TLS_DEGs. (**D**) Venn diagram showing the intersection of TLS_DEG and TLS_COX genes. (**E**) Heatmap displaying the expression of CXCL13, CXCL10, CXCL9, and CXCL11 genes in ovarian cancer. (**F**, **G**) Survival analysis and differential expression of CXCL13, CXCL9, CXCL10, and CXCL11 genes in ovarian cancer.

The workflow of this study is illustrated in Figure 1. A total of 14 TLS genes exhibited differential expression between the tumor and normal groups. With the exception of CCL21, all other genes were upregulated in the tumor group (Figure 2A). Protein interaction networks revealed that several genes, including CCL21, CCL2, CXCL10, CCL5, CXCL13, and CCL8, exhibited close interactions with other genes (Figure 2B). Univariate Cox analysis demonstrated that CXCL9, CXCL10, CXCL11, and CXCL13 were protective factors in ovarian cancer (HR < 1, p < 0.01, Figure 2C). Subsequently, we intersected the differentially expressed TLS genes with these four genes, indicating that these four genes were both differentially expressed and prognostically protective (Figure 2D). Heatmaps displayed high expression of these four genes in the tumor group (Figure 2E). Kaplan-Meier curves indicated that patients with high expression had a more favorable prognosis (p < 0.001, Figure 2F). In the Imvigor cohort, the effective treatment group showed elevated expression of CXCL9, CXCL10, CXCL11, and CXCL13 compared to the treatment-resistant group.

### Non-negative matrix factorization (NMF) analysis and further analysis

**Figure 3:**
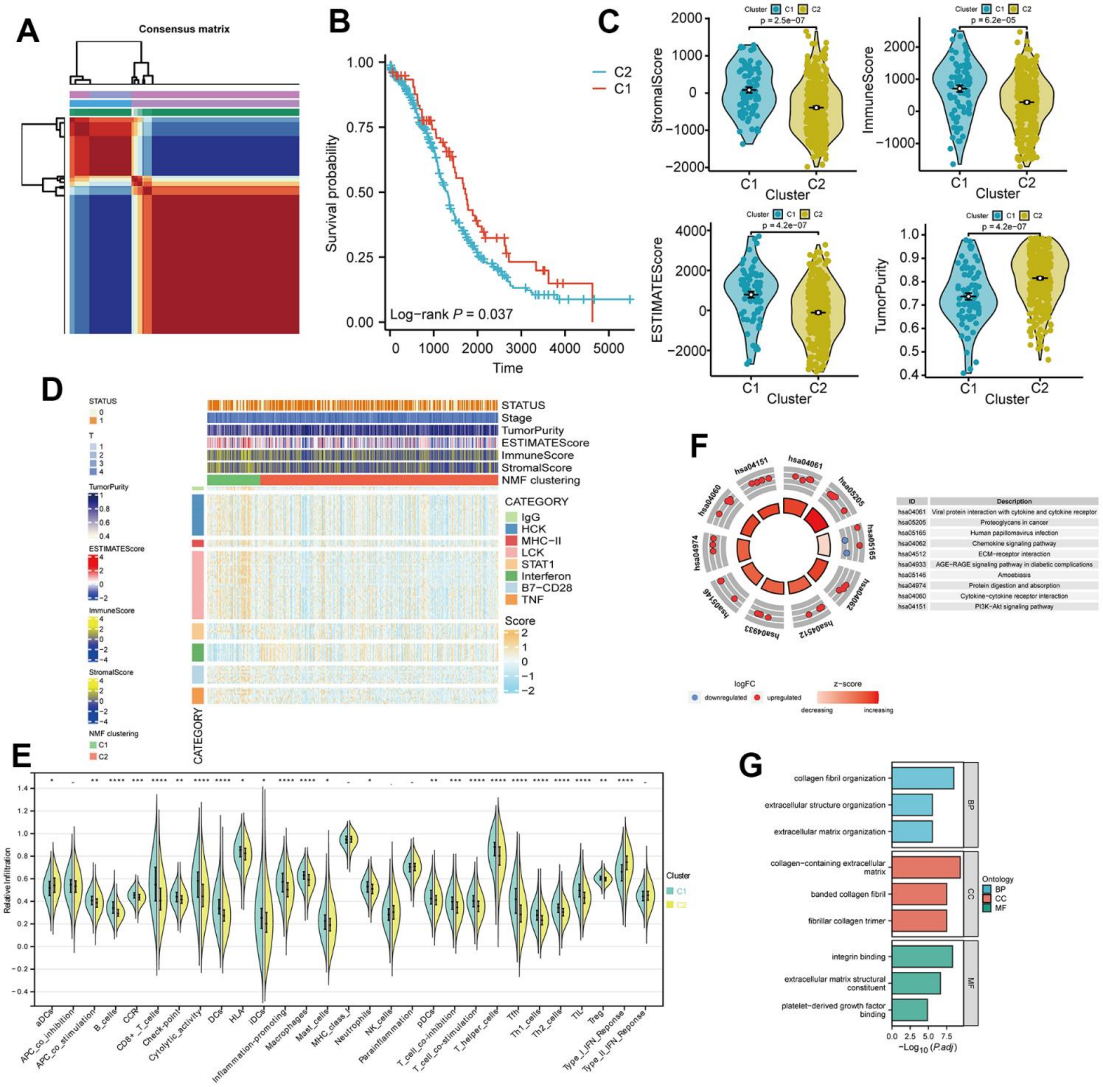
**Non-negative matrix factorization (NMF) subtyping of the training set and functional analysis.** (**A**) Consensus plot showing the classification of patients into two clusters (C1 and C2) using NMF analysis. (**B**) Kaplan-Meier survival curve comparing the two clusters (C1 and C2). (**C**) Comparison of StromalScore, ImmuneScore, and ESTIMATEScore of C1 and C2. (**D**) Heatmap of the two clusters with respect to immune inflammation. (**E**) Enrichment scores of 29 immune cells in the two clusters. (**F**) Kyoto Encyclopedia of Genes and Genomes (KEGG) pathway analysis of differentially expressed genes between the two clusters. (**G**) Gene Ontology (GO) analysis results, classifying the genes into Biological Process (BP), Cellular Component (CC), or Molecular Function (MF) categories.

In accordance with the principle of “high cohesion, low coupling,” we categorized TCGA-OV patients into 2 clusters (Figure 3A). Kaplan-Meier (KM) curves indicated that patients in cluster 1 had a better prognosis (p = 0.037, Figure 3B). TIME (Tumor Immune Microenvironment) analysis revealed that cluster 1 had higher Immune Score, Stromal Score, and Estimate Score, whereas cluster 2 exhibited higher tumor purity (Figure 3C). The heatmap illustrated higher survival rates among patients in cluster 1. IgG, HCK, MHC-I I, LCK, B7-CD28, and TNF were expressed at higher levels in cluster 1, whereas STAT1 and Interferon were expressed at higher levels in cluster 2 (Figure 3D). Immune analysis demonstrated significant differences in the infiltration of various immune cells between the 2 clusters (p < 0.05). The immune cell infiltration scores in cluster 1 were notably higher than those in cluster 2, consistent with the TIME results (Figure 3E). Furthermore, we conducted differential gene expression analysis for the 2 clusters (Supplementary Table 2). KEGG pathway analysis of the differentially expressed genes indicated upregulation in pathways related to protein polysaccharides, chemokine signaling, ECM-receptor interaction, cytokine-cytokine receptor interaction, and PI3K-Akt signaling in cancer (Figure 3F). GO analysis revealed upregulation of differentially expressed genes in various extracellular matrix components and cellular constituents (Figure 3G).

### Construction and validation of a prognostic model based on TLS gene-associated lncRNA

**Figure 4:**
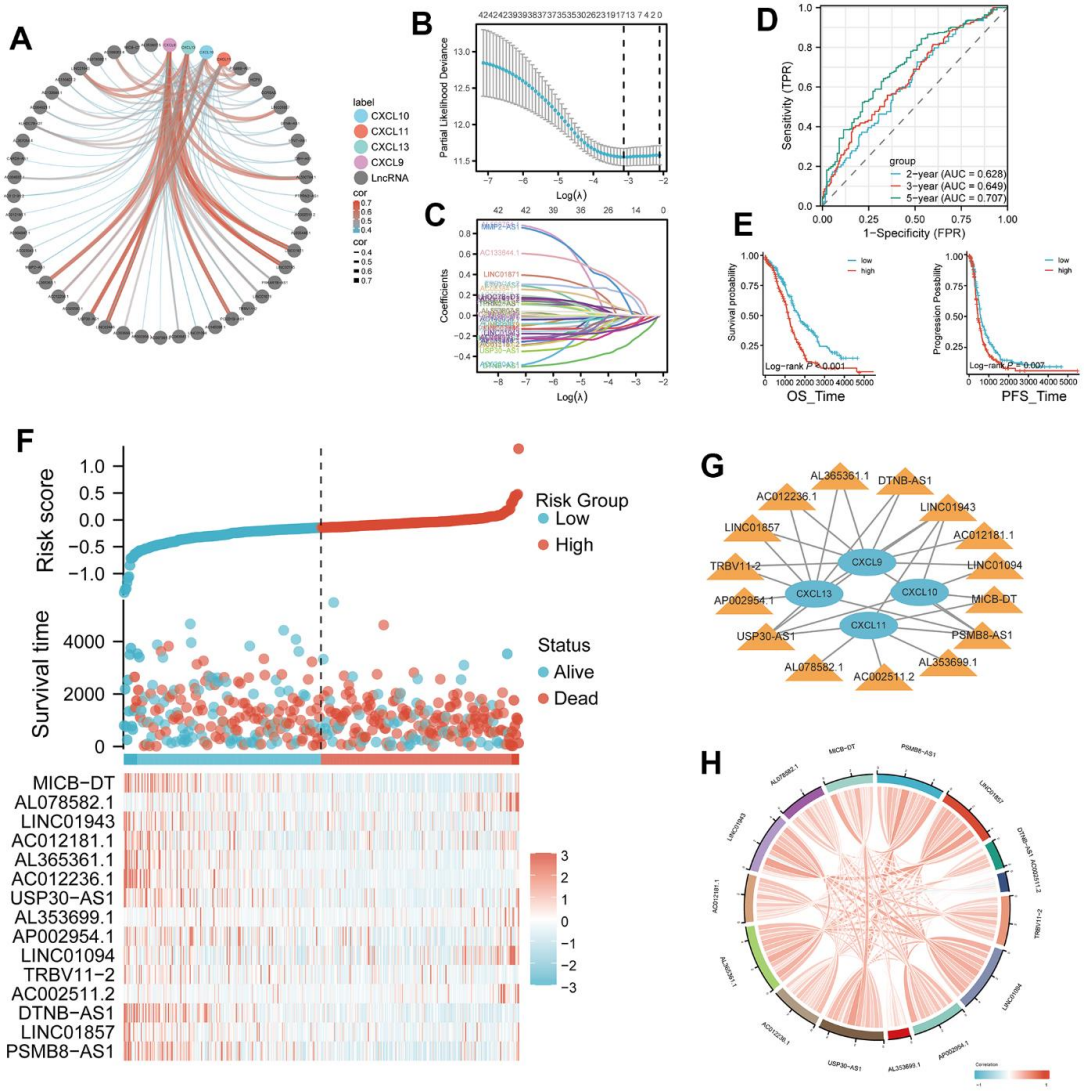
**Development of a four-gene risk model and its correlation with lncRNA.** (**A**) Correlation analysis between CXCL9, CXCL10, CXCL11, and CXCL13 and lncRNA (cor=0.35, p < 0.05). (**B**, **C**) Least Absolute Shrinkage and Selection Operator (LASSO) regression analysis of the 42 differentially expressed mRNAs (DEMs), with 16 selected as valuable prognostic indicators. (**D**) Receiver operating characteristic (ROC) curve of the four-gene model at 2, 3, and 5 years. (**E**, **F**) Comparison of overall survival (OS) and progression-free survival (PFS) between the high- and low-risk groups. (**G**) Visualization of the associations between the four genes of interest (CXCL9, CXCL10, CXCL11, and CXCL13) and 15 lncRNAs using Cytoscape. (**H**) Chord plot showing correlations between the four genes and the 15 model lncRNAs.

Based on the transcriptome data of TCGA-OV tumor patients, we conducted an analysis of long non-coding RNAs (lncRNAs) co-expressed with TLS genes, as obtained from the Venn diagram intersection in Figure 4A. Spearman analysis revealed a strong positive correlation (p < 0.001, R > 0.5, Supplementary Figure 1) between CXCL9, CXCL10, CXCL11, CXCL13, and their respective top 4 correlated lncRNAs. By employing LASSO analysis, we derived a 15-gene risk model (MICB-DT, AL078582.1, LINC01943, AC012181.1, AL365361.1, AC012236.1, USP30-AS1, AL353699.1, AP002954.1, LINC01094, TRBV11-2, AC002511.2, DTNB-AS1, LINC01857, PSMB8-AS1) (Figure 4B, 4C, and Supplementary Table 3). Univariate Cox analysis demonstrated that 13 lncRNAs significantly impacted patient prognosis, with the majority serving as protective factors. This observation was further corroborated by KM survival curves (Supplementary Figure 2). ROC curves indicated excellent diagnostic performance of the model at 2, 3, and 5 years (AUC > 0.6, Figure 4D). Patients in the high-risk group exhibited worse overall survival (OS) and progression-free survival (PFS) (p < 0.01, Figure 4E). Risk factor analysis illustrated an increasing number of deceased patients and a gradual reduction in the expression of model lncRNAs with rising risk scores (Figure 4F). There was a wide-ranging association between CXCL9, CXCL10, CXCL11, CXCL13, and the model lncRNAs (Figure 4G). Additionally, strong positive correlations were observed among the model lncRNAs themselves (Figure 4H).

### Establishment and validation of the nomogram prognostic model

**Figure 5:**
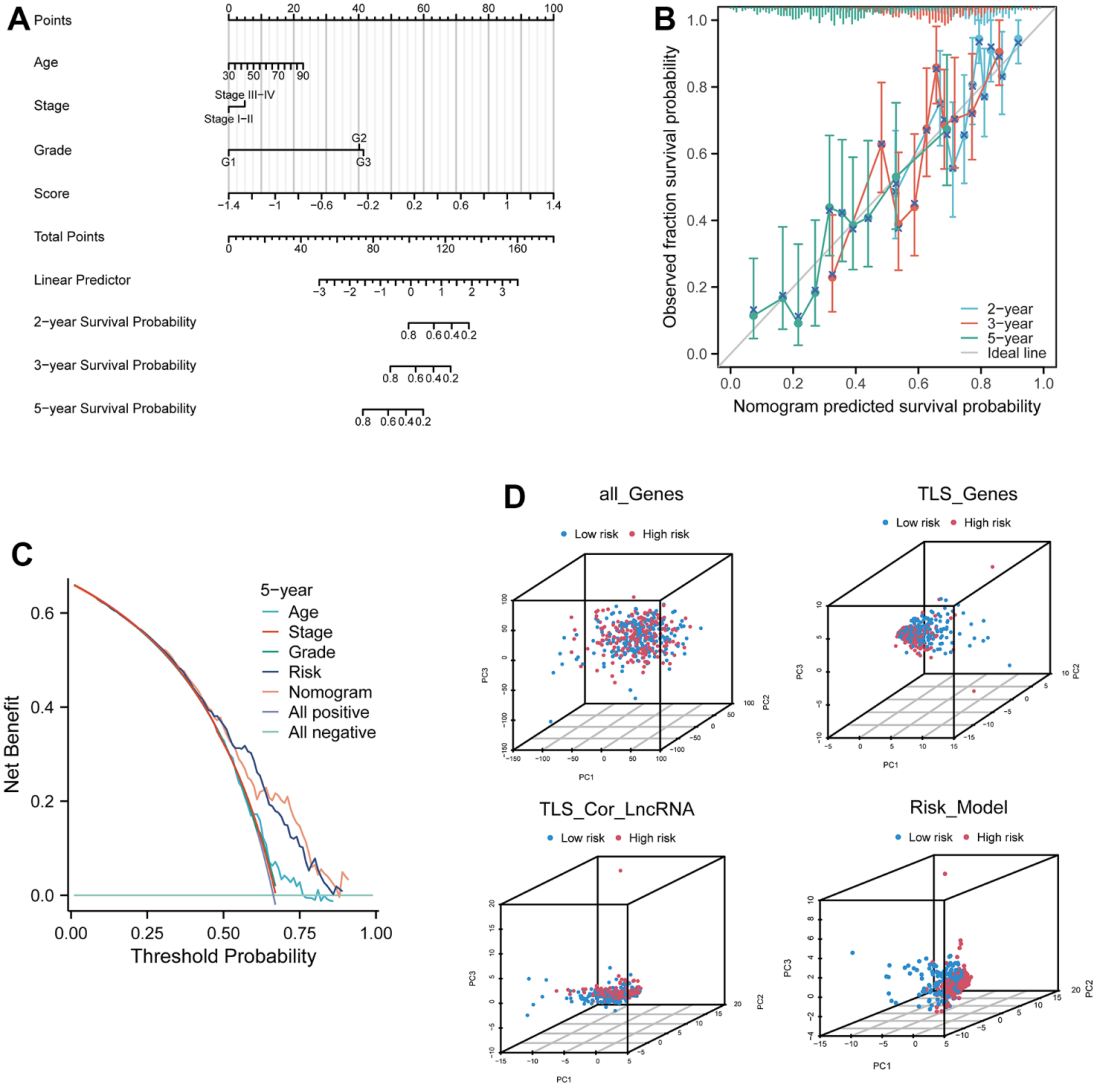
**Prognostic model based on four related genes and clinical pathological factors.** (**A**) Nomogram combining the expression of CXCL9, CXCL10, CXCL11, and CXCL13 genes and independent clinical risk factors (age and pathological stage). (**B**) Calibration plot for the nomogram, showing good agreement between predicted and observed results. (**C**) Decision curve analysis (DCA) curve comparing the established ovarian cancer (OV) nomogram and classical TNM tumor staging. (**D**) Three-dimensional principal component analysis plots for all genes, TLS genes, TLS-related LncRNA genes, and model Lnc, showing improved separation.

In our study, we incorporated age, stage, grade, and risk score to comprehensively construct a nomogram model for predicting patients' 2-year, 3-year, and 5-year survival outcomes (Figure 5A, and Supplementary Table 4). Calibration curves demonstrated the excellent predictive accuracy of the nomogram at all three time points (Figure 5B). Decision curve analysis (DCA) curves further indicated that the nomogram model yielded superior decision-making benefits (Figure 5C). Moreover, the results of a three-dimensional principal component analysis (3D PCA) revealed that under the context of lncRNA in the model, there was better differentiation between high-risk and low-risk groups of samples (Figure 5D).

### Multiple analytical modes reveal the underlying mechanisms of TLS-related lncRNAs

**Figure 6:**
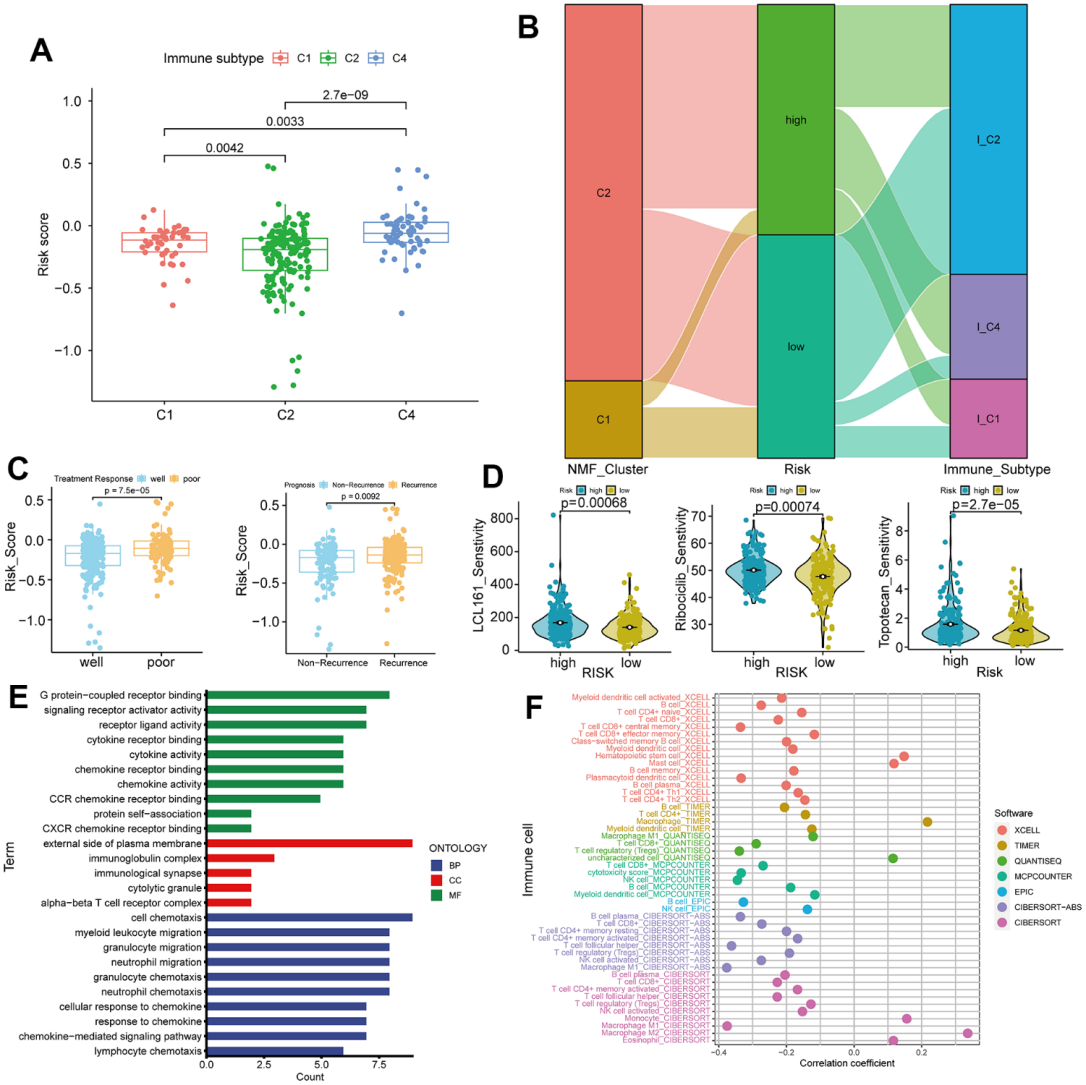
**Immune subtypes, treatment response, and gene ontology (GO) analysis in high and low-risk groups.** (**A**) Differences in risk scores among C1, C2, and C4 immune subtypes. (**B**) Sankey plot showing high and low expression trends of different clinical features of C1, C2, and C4 subtypes in samples of patients with ovarian cancer. (**C**) Comparison of risk scores in treatment response (Well vs. Poor) and recurrence (Recurrence vs. Non-Recurrence). (**D**) Drug sensitivity analysis for LCL161, ribociclib, and topotecan treatments in ovarian cancer. (**E**) GO analysis in high and low-risk groups. (**F**) Correlations between tumor immunity and ovarian cancer using data from various databases.

The risk scores among TCGA immune subtypes exhibit significant differences (p < 0.0001, Figure 6A). The Sankey plot illustrates that a majority of patients in cluster 2 occupy both risk groups, while cluster 1 patients are in the minority. Patients in the low-risk group are primarily distributed within immune subtype 2, whereas those in the high-risk group are scattered across various immune subtypes (Figure 6B). The ineffective treatment group shows higher risk scores (p = 7.5e-05), and the recurrence group exhibits even higher risk scores (p = 0.0092, Figure 6C). Sensitivity analysis of 200 drugs (Supplementary Table 5) reveals that patients in the high-risk group are more sensitive to three drugs, LCL161, Ribociclib, and Topotecan, compared to the low-risk group (p < 0.0001, Figure 6D). These drugs are currently under investigation, and our results suggest their potential in ovarian cancer treatment. Gene Ontology (GO) analysis of differentially expressed genes in the two risk groups indicates that neutrophil and lymphocyte functions are prominent in biological processes (BP). Plasma cell function ranks highest in cellular components (CC), while chemotaxis tops molecular functions (MF). This underscores the significance of tumor immunity as a crucial factor distinguishing high and low-risk groups (Figure 6E). Analysis of eight immune cell infiltrations reveals significant differences in immune cell scores between high and low-risk groups, implying a strong association between the lncRNA risk formula based on TLS and various immune cells (Figure 6F).

**Figure 7:**
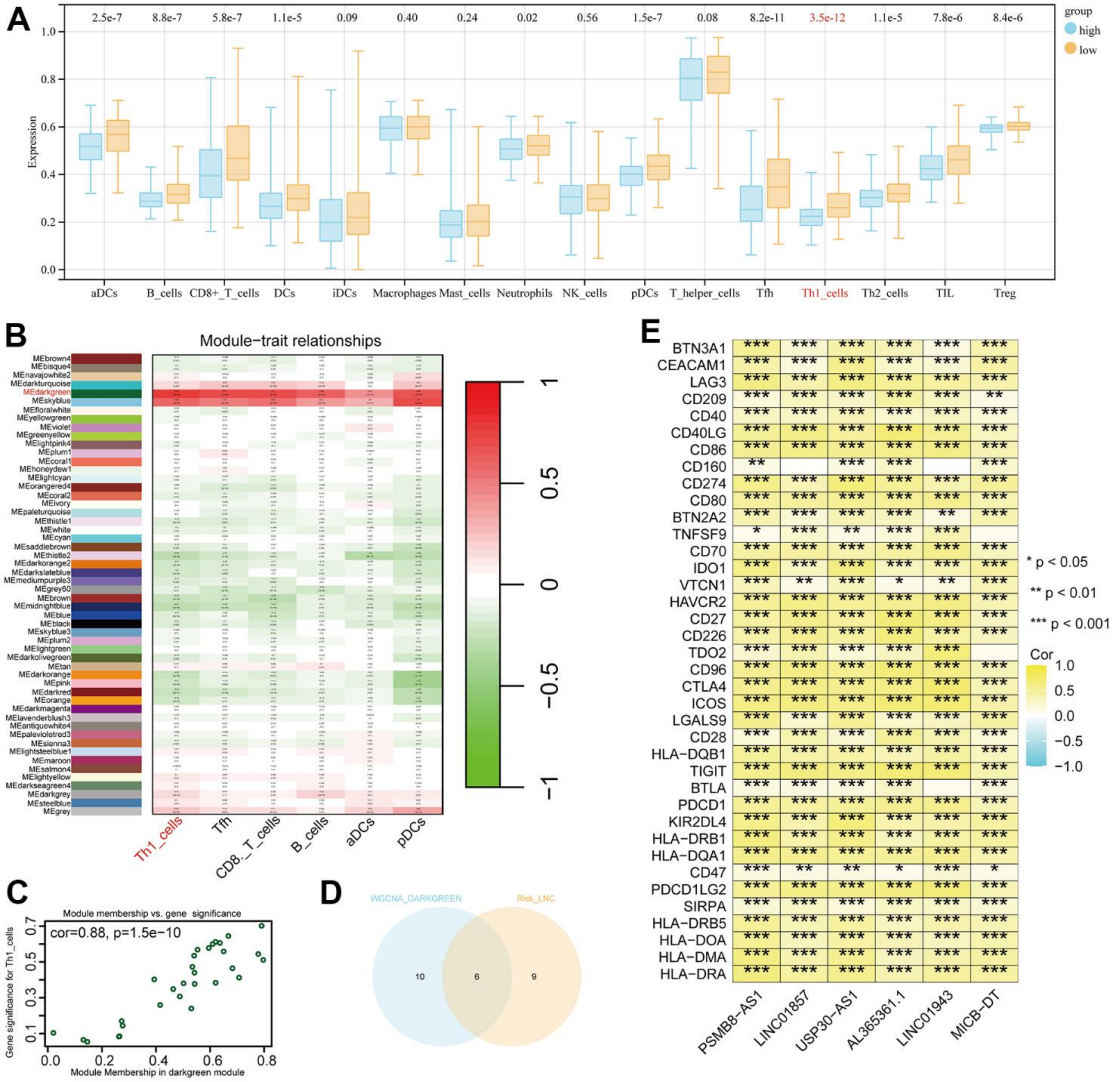
**Differential immune cells between high and low-risk groups and Th1-related LncRNAs.** (**A**) Most significant difference in Th1_cells between high and low-risk groups. (**B**) Weighted correlation network analysis (WGCNA) results showing the dark-green module as the most related to Th1. (**C**) Identification of 16 lncRNAs with a correlation coefficient > 0.4. (**D**) Venn diagram intersection of Th1-related lncRNA and model genes, identifying six genes with Th1 correlation and strong prognostic prediction ability. (**E**) Co-expression relationships between the six genes and immune checkpoints (P < 0.05).

In the low-risk group, most immune cell infiltrations are higher than those in the high-risk group, with the most significant difference observed in Th1 cells (p = 3.5e-12, Figure 7A). We conducted a correlation analysis between the six immune cells with the smallest p-values and various modules of WGCNA. Darkgreen module exhibits the highest correlation with Th1 (cor = 0.79, p = 9e-89, Figure 7B, 7C). We selected 16 lncRNAs with correlation coefficients greater than 0.4 from this module and identified six genes that intersect with model genes, demonstrating strong prognostic prediction capabilities related to Th1 (Figure 7D). The co-expression relationship between these six intersected genes and immune checkpoints is significant (Figure 7E).

**Figure 8:**
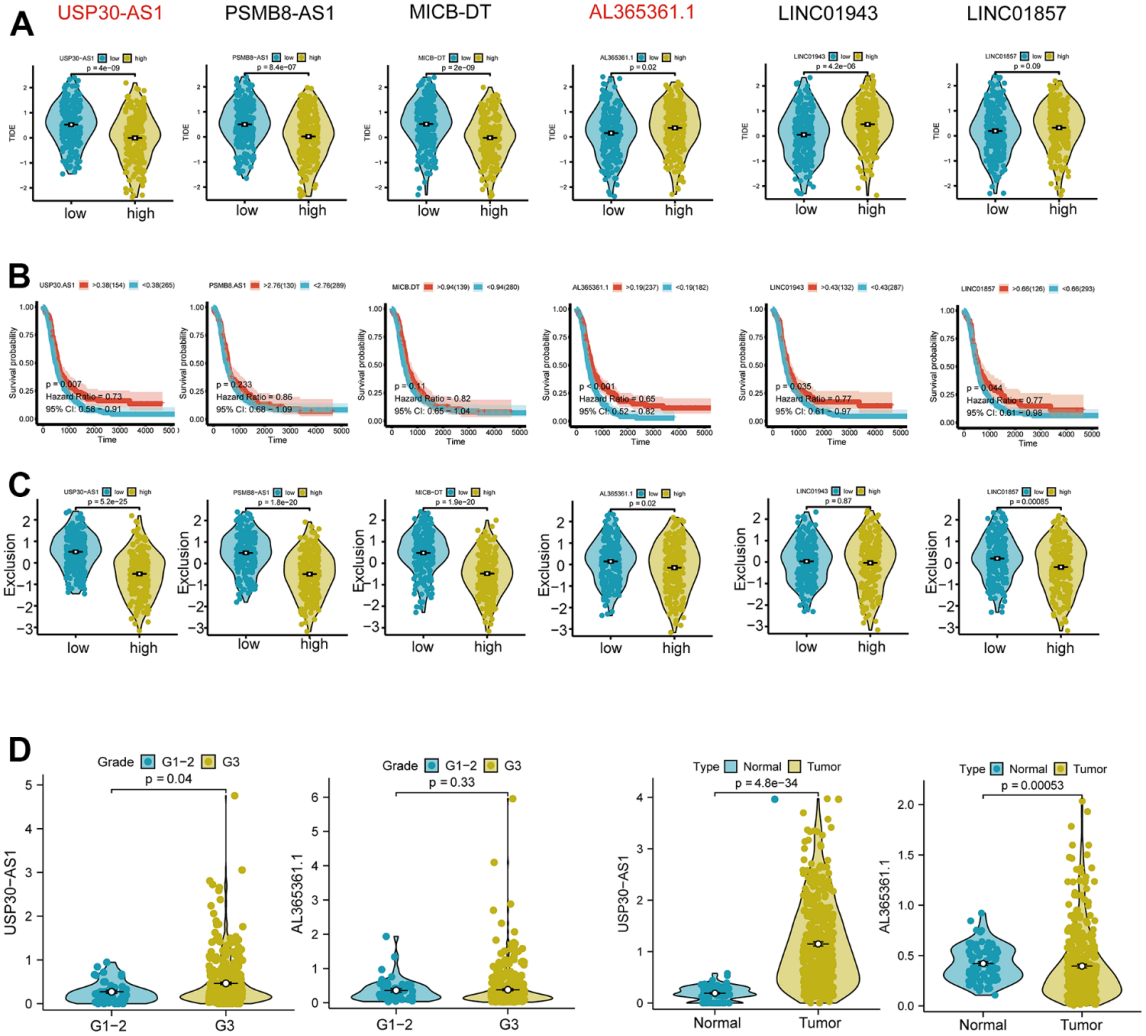
**Differential expression and functional analysis of the six Th1-related genes.** (**A**) Tumor immune dysfunction and exclusion (TIDE) score analysis showing significant differential expression of USP30-AS1, PSMB8-AS1, MICB-DT, AL365361.1, and LINC01943 genes in tumors, while the score of LINC01857 is not significant. (**B**) Survival probability analysis showing a significant difference in the expression of the USP30-AS1 gene. (**C**) Exclusion analysis showing significant expressions of USP30-AS1, PSMB8-AS1, MICB-DT, AL365361.1, and LINC01857 genes. (**D**) Grade and type grouping analysis revealing significant differences in the expression of USP30-AS1 gene.

**Figure 9:**
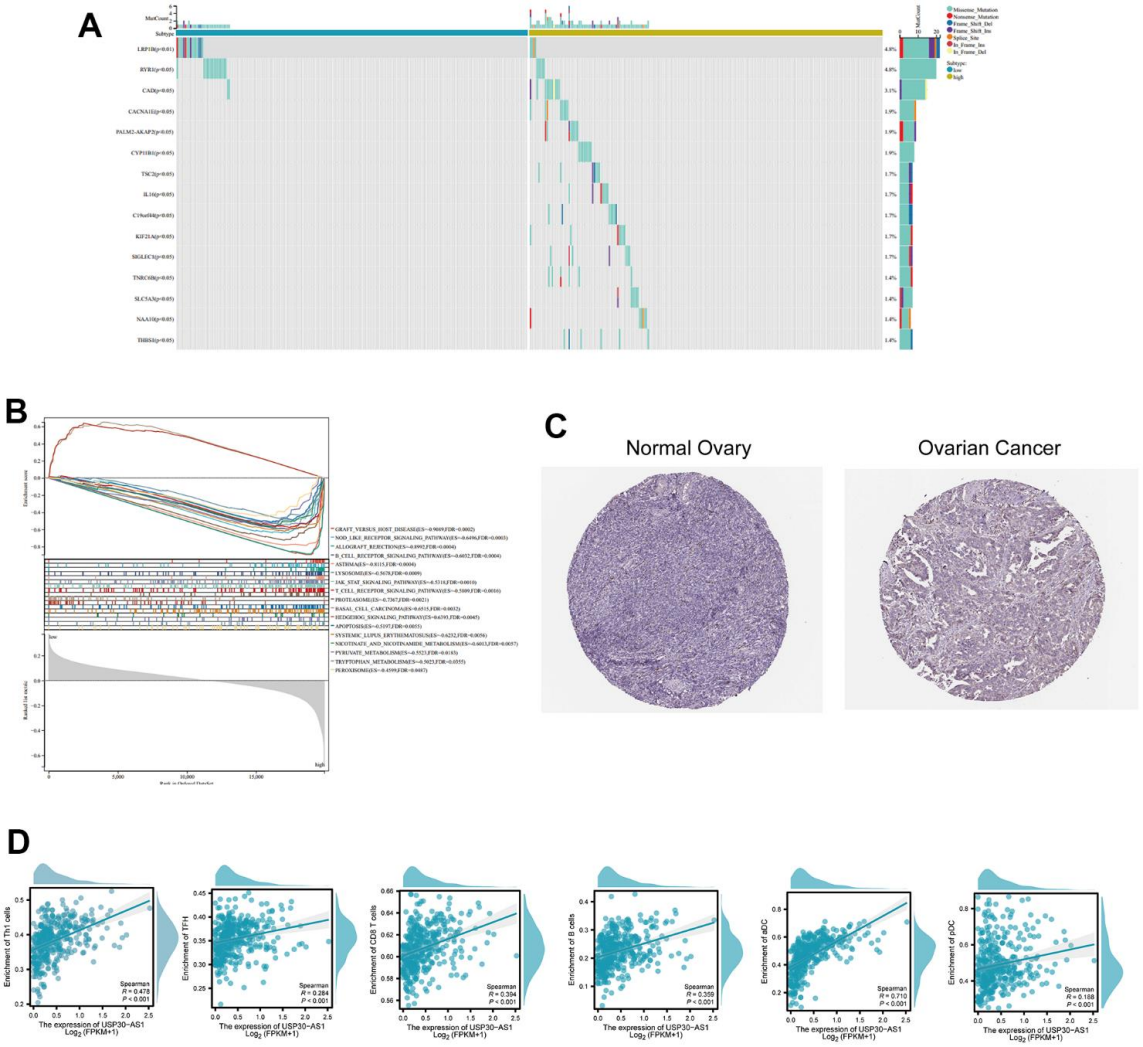
**Differential expression and functional analysis of the USP30-AS1 gene in ovarian cancer.** (**A**) Identification of 15 most significantly mutated genes in high and low expressing groups of USP30-AS1. (**B**) Gene Set Enrichment Analysis (GSEA) of USP30-AS1 gene in high and low expression groups. (**C**) Immunohistochemical images of the LRP1B gene in normal and tumor tissue from the HPA database (HPA069094). (**D**) Correlation analysis between USP30-AS1 gene expression and various immune cells, suggesting a potential role in immune cell infiltration regulation in ovarian cancer.

Subsequent analysis focused on the six intersected genes. We employed TIDE scoring, PFS survival curves, and TIDE Exclusion scoring to screen out USP30-AS1 and Al365261.1 (Figure 8A–8C). USP30-AS1 exhibited differences between Grade 2 subgroups, while Al365261.1 did not. Consequently, we decided to proceed with the analysis of USP30-AS1 (Figure 8D). We categorized the expression of USP30-AS1 into high and low expression groups based on its median expression value. We analyzed the mutation differential genes between the two groups and presented the top 15 significant genes in a waterfall plot. LRP1B exhibited the highest mutation level (Figure 9A). Multiple GSEA analyses of the two expression groups indicated the involvement of RYR1 and CACNA1E in the calcium channel pathway: CALCIUM SIGNALING PATHWAY. This suggests that USP30-AS1 may interfere with the tumor immune response of immune cells such as Th1 by affecting the physiological opening of calcium channels, thereby promoting/inhibiting tumor immune escape (Figure 9B). Immunohistochemical analysis demonstrated higher expression of LRP1B in tumor tissues (Figure 9C). The infiltration of multiple immune cells is positively correlated with the expression of USP30-AS1 (R > 0.2, p < 0.001, Figure 9D).

### Pan-cancer analysis of USP30-AS1

The results of the differential expression analysis indicate that USP30-AS1 exhibits differential expression across multiple cancer cohorts, except for uterine carcinosarcoma (UCS), pheochromocytoma and paraganglioma (PCPG), adrenocortical carcinoma (ACC), and kidney chromophobe (KICH). In most cancer types, USP30-AS1 is upregulated (P < 0.05, Figure 9A). From a macroscopic perspective, immune cell infiltration scores are inversely correlated with USP30-AS1 expression in most cancers. However, in the case of immune cells such as aDC, CD8^+^ T cells, Macrophages M1, and USP30-AS1 expression, a positive correlation is observed in most cancer cohorts (Figure 9B). Given that the differential analysis of immune cells between high and low-risk groups previously revealed the most significant statistical difference in Th1 cells between the two risk groups, we have selected the four cancer cohorts with the highest absolute correlation coefficients between Th1 cell infiltration scores and USP30-AS1 expression for scatter plot representation of their correlation. In uterine corpus endometrial carcinoma (UCEC), head and neck squamous cell carcinoma (HNSC), and Cervical squamous cell carcinoma (CESC), Th1 cell infiltration positively correlates with USP30-AS1 expression (P < 0.0001, correlation coefficient >0.3). Conversely, in glioblastomas (GBM), Th1 cell infiltration exhibits a negative correlation with USP30-AS1 expression (P < 0.0001, correlation coefficient = -0.36, Figure 9C).

### USP30-AS1 is highly expressed in ovarian cancer cell lines

**Figure 10:**
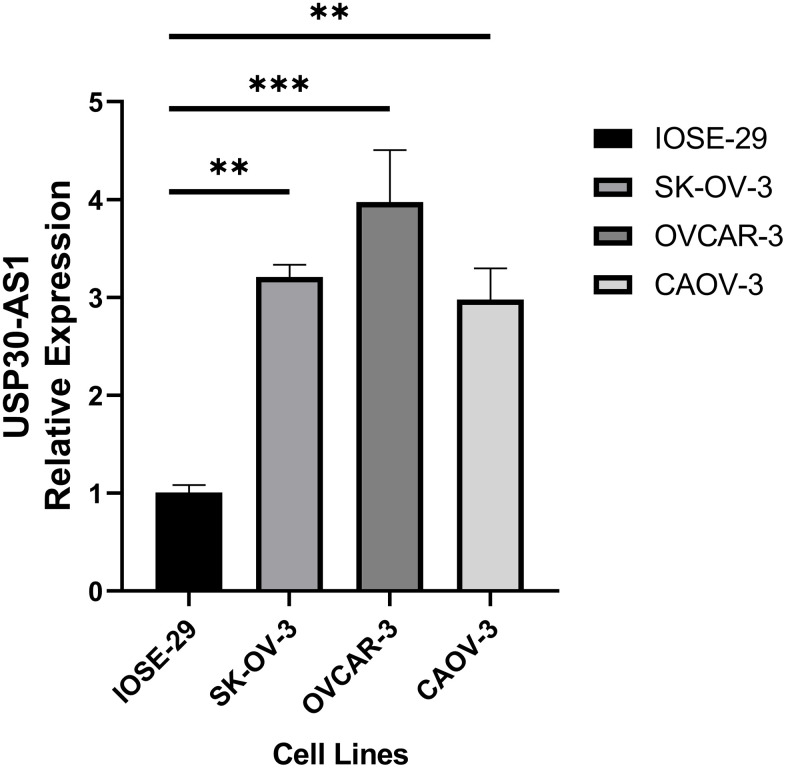
RT-qPCR analysis of ovarian cancer cell line and normal ovarian cell line.

We conducted RT-qPCR analysis on three ovarian cancer cell lines and one normal ovarian epithelial cell line. The results revealed a significantly higher expression of USP30-AS1 in the ovarian cancer cell lines compared to the normal ovarian epithelial cell line (p < 0.01). Among them, the OVCAR-3 cell line exhibited the highest expression level (Figure 10). Hence, we conclude that USP30-AS1 is highly expressed in ovarian cancer cell lines.

## DISCUSSION

This study aimed to find the underlying implications of the USP30-AS1 gene in the prognosis of ovarian cancer, and examine its potential influence on the infiltration of immune cells. We found a significant association of USP30-AS1 expression with Th1_cells, TFH, CD8_T cells, B cells, aDC, and pDC cells. These observations underscore the possible regulatory function of USP30-AS1 in governing immune activities within the TIME and its potential impact on ovarian cancer treatment responses and prognosis. The findings offer novel insights into the complicated correlations between the tumor and the immune system, paving the way for the development of precision immunotherapeutic interventions in cancer treatment. Our finding adds to the growing pool of proof suggesting the vital function of lncRNAs in terms of the emergence and advancement of cancer. Earlier studies have linked several lncRNAs to the control of immune cell penetration in TIME. For instance, LINC01943, another lncRNA scrutinized in our research, has been documented to govern the entry of CD8^+^ T cells and natural killer cells in non-small cell lung cancer [33]. Likewise, studies have also indicated that LINC01857 regulates the penetration of immune cells like macrophages and Tregs in stomach cancer [34]. Our discoveries about USP30-AS1 underline the importance of lncRNAs in cancer prognosis.

The direct link seen between USP30-AS1 levels and Th1 cells, TFH, CD8 Tcells, and Bcells suggests its potential role in creating an active TME. The presence of these immune cells is widely linked with a favorable prognosis in various malignancies, such as ovarian cancer [35–37]. For example, increased infiltration of Th1 lymphocytes is associated with more favorable survival results among ovarian cancer patients [38]. Likewise, the existence of CD8^+^ T lymphocytes and B lymphocytes in TIME has been associated with better predictive outcomes in ovarian cancer. Our discoveries conform with these prior findings, implying that USP30-AS1 could have significant predictive consequences in ovarian cancer [39, 40].

An intriguing observation was made in this study wherein we discovered a positive association of the expression levels of USP30-AS1 with aDC and pDC. Both aDCs and pDCs are involved in tumor immunity, with aDCs primarily being essential in activating T-cell responses, thereby mitigating the tumor growth [41]. The positive association between USP30-AS1 expression and aDCs plays a paramount role for USP30-AS1 in promoting T-cell activation, which could contribute to improved prognosis. We also conducted a pan-cancer analysis of USP30-AS1. USP30-AS1 exhibits upregulation in the majority of cancers and demonstrates a significant correlation with various immune cell infiltrations. The infiltration of immune cells such as aDC, CD8^+^ T cells, Macrophages M1, and USP30-AS1 expression shows a positive correlation in most cancers, consistent with our previous findings. We hypothesize that USP30-AS1 may play a crucial role in multiple cancers and potentially exerts its pro-cancer effects through close interactions with immune cells. Further in-depth research is warranted to validate our hypothesis. However, the role of pDCs in cancer remains debatable, with some studies reporting pro-tumorigenic functions and others suggesting anti-tumorigenic roles [42, 43]. Our findings warrant further investigation into the functional significance of the observed relationship of USP30-AS1-pDC in ovarian cancer. We conducted RT-qPCR analysis on ovarian cancer cell lines and normal ovarian cell lines, revealing that USP30-AS1 is highly expressed in ovarian cancer cell lines as compared to normal ovarian cell lines. The results of our wet experiment confirmed the conclusion of our analysis.

Comparing our findings with previous studies on USP30-AS1, we observed some similarities and differences. Li et al. The research conducted by Li revealed aberrant USP30-AS1 expression in colorectal cancer, with a marked increase in levels. Interestingly, this increased expression apparently correlated with the promotion of several aggressive processes, including enhanced cancer cell proliferation, migration, and invasion. These findings also strongly suggest a potential pivotal role of USP30-AS1 in the neoplastic progression of colorectal cancer by promoting the malignant behaviors of cancer cells, thereby suggesting an undeniable pro-tumorigenic function of USP30-AS1 in this setting [44]. In contrast, our current study provides significant insights into the potential mechanisms by which USP30-AS1 may contribute to ovarian cancer tumorigenesis. We discovered that this lncRNA is capable of modulating immune cell infiltration within the TIME, and profoundly impact the progression and prognosis of the tumor. Our investigations indicate that the overexpression of USP30-AS1 is positively associated with increased infiltration of Th1 and dendritic cells within the TME, which in turn is directly associated with improved prognoses of ovarian cancer patients. This novel finding suggests the potentially vital role of USP30-AS1 in shaping the TME by regulating the local immune response. These discoveries may pave the way for novel therapeutic approaches that target this lncRNA, to enhance host immune responses against ovarian cancer cells and ultimately improve patient outcomes. These discrepancies could be attributed to the tissue-specific functions of lncRNAs or differences in the underlying molecular mechanisms in colorectal and ovarian cancers. To gain further insight into the tumor biology of various cancer types, studies in the near future are aiming to delineate the underlying mechanisms by which USP30-AS1 regulates pathological processes, such as cancer cell proliferation, migration, and invasion. It is also imperative to further investigate the potential tissue-specificity of USP30-AS1 functions in the context of cancer development and elucidate how USP30-AS1 may affect distal signaling pathways and genomic regulatory networks. Such research may provide novel avenues for identifying specific markers for cancer prognosis and developing targeted therapies against various malignant neoplasms.

Our study has several limitations that should be acknowledged. First, our analysis was based on bioinformatics and publicly available datasets. While the findings of our study indicate a promising role of USP30-AS1 in modulating immune cell infiltration within the TIME, we acknowledge that the potential role of this lncRNA cannot be fully understood based on our current research. The complex interactions between USP30-AS1 and immune cells within the TME require extensive and more comprehensive investigations for a deeper understanding of their mechanistic underpinnings. Further studies combining different methodologies, such as transcriptomics, proteomics, and functional assays, will aid in thoroughly understanding the molecular and cellular mechanisms of USP30-AS1 in tumor-immune crosstalk and TME modulation. These findings can provide invaluable insights into the potential clinical applications of USP30-AS1 modulation for precision immune-oncology approaches in cancer management. Our findings need to be confirmed in the future studies through experimental validation using *in vitro* and *in vivo* models. Second, the functional roles of USP30-AS1 in immune cell infiltration and its downstream molecular mechanisms were not explored in this study, and need to be investigated to elucidate the molecular pathways through which USP30-AS1 modulates immune cell infiltration in ovarian cancer.

Despite certain limitations, our research presents novel insights on the underlying role of USP30-AS1 in ovarian cancer progression and immune modulation. The study findings indicate that the aberrant expression of USP30-AS1 may hold promise as a distinctive prognostic marker for ovarian cancer, with significant therapeutic implications. Additional research is required to elucidate the molecular mechanisms underpinning the relationship between USP30-AS1 and the immune system and to validate these observations in varied independent patient cohorts and experimental models. These future studies could improve our understanding of the molecular circuits regulated by USP30-AS1, eventually allowing for innovative treatments against ovarian cancer.

To conclude, our study offers crucial insights into the likely role of USP30-AS1 in determining prognostic features of ovarian cancer and its potential impact on the TIME. Our research effectively demonstrates the positive association between USP30-AS1 expression levels and several types of immune cells, like Th1 cells, suggesting a vital role of this gene in shaping the immune landscape in ovarian carcinoma. Overall, our findings contribute to the ever-increasing pool of evidence indicating the significance of lncRNAs in the context of cancer immunity and highlights the urgent need of studies investigating the functional significance of USP30-AS1 in modulating immune responses in ovarian cancer.

## Supplementary Material

Supplementary Figures

Supplementary Table 1

Supplementary Table 2

Supplementary Table 3

Supplementary Table 4

Supplementary Table 5
