# Supplementary material for: Prognostic role of long non-coding RNA USP30-AS1 in ovarian cancer: insights into immune cell infiltration in the tumor microenvironment
Source: Aging (Albany NY). 2023 Dec 4;15(23):13776–98. doi: 10.18632/aging.205262 (PMC10756134; doi:10.18632/aging.205262)
Supplement: Supplementary Figures [file aging-15-205262-s001.pdf]

SUPPLEMENTARY FIGURES

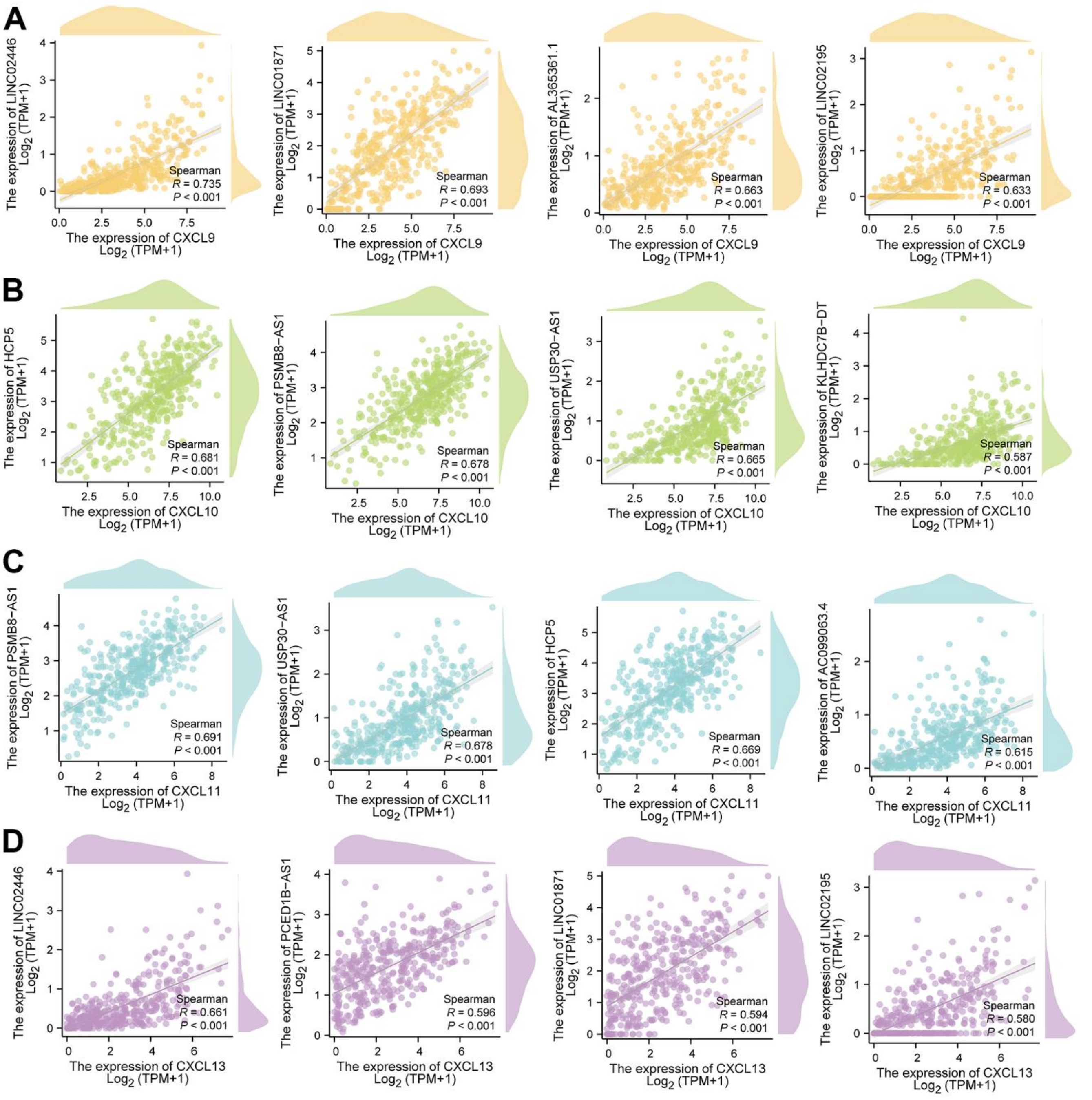

Supplementary Figure 1. Correlation scatter plots of CXCL9, CXCL10, CXCL11 and CXCL13 with the four lncRNAs with the highest correlation degree, respectively. (**A**) Correlation scatter plot of CXCL9 and the 4 lncRNAs with the highest correlation degree. (**B**) Correlation scatter plot of CXCL10 and the 4 lncRNAs with the highest correlation degree. (**C**) Correlation scatter plot of CXCL11 and the 4 lncRNAs with the highest correlation degree. (**D**) Correlation scatter plot of CXCL13 and the 4 lncRNAs with the highest correlation degree.

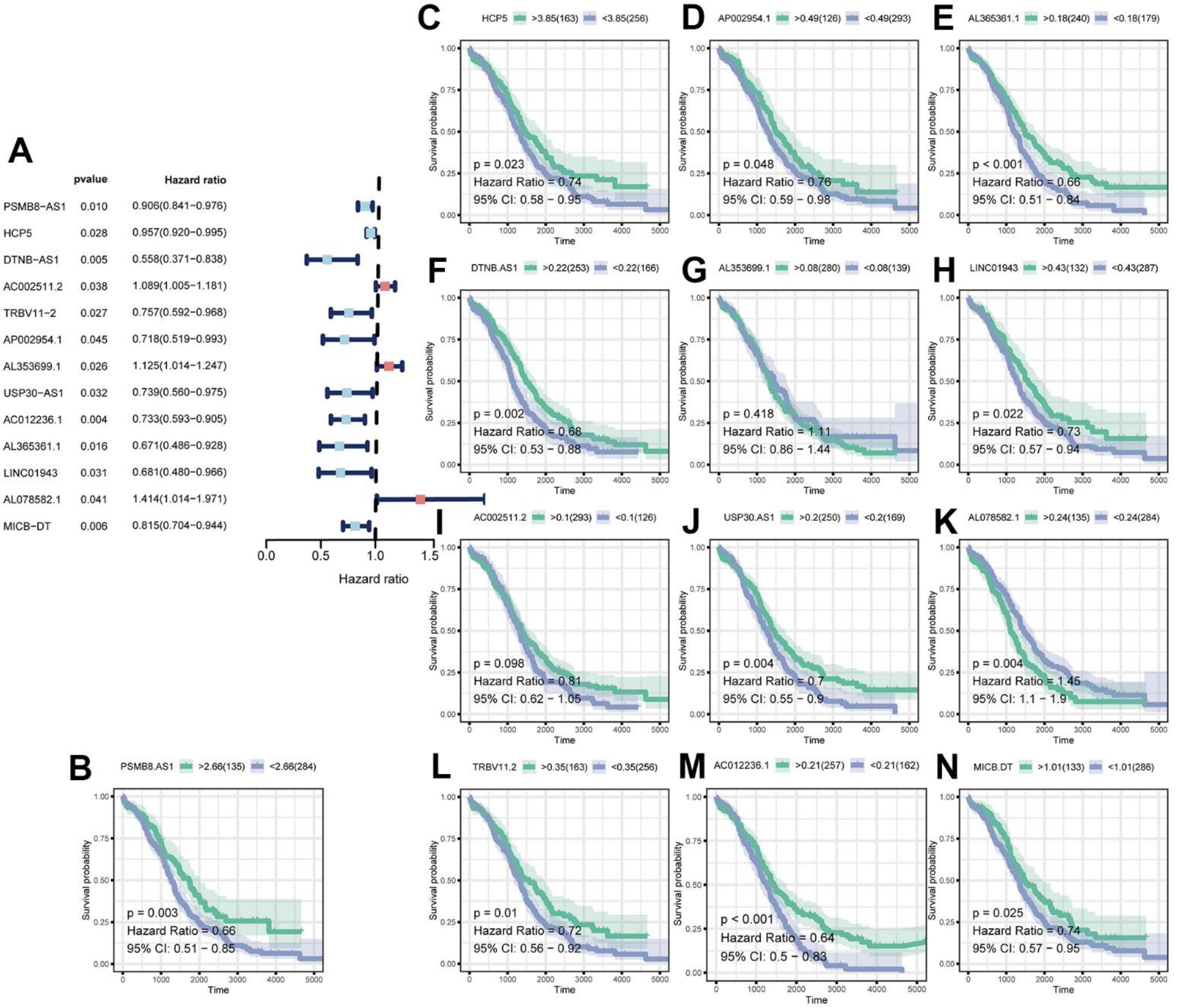

Supplementary Figure 2. Forest plot and survival curve of 13 lncRNAs. (**A**) Forest plot of 13 lncRNAs. (**B**) Survival curve of PSMB8-AS1. (**C**) Survival curve of HCP5. (**D**) Survival curve of AP002954.1. (**E**) Survival curve of AL365361.1. (**F**) Survival curve of DTNB.AS1. (**G**) Survival curve of AL353699.1. (**H**) Survival curve of LINC01943. (**I**) Survival curve of AC002511.2. (**J**) Survival curve of USP30AS1. (**K**) Survival curve of AL078582.1. (**L**) Survival curve of TRBV11.2. (**M**) Survival curve of AC012236.1. (**N**) Survival curve of MICB.DT.
