# Supplementary material for: Prognostic role of long non-coding RNA USP30-AS1 in ovarian cancer: insights into immune cell infiltration in the tumor microenvironment
Source: Aging (Albany NY). 2023 Dec 4;15(23):13776–98. doi: 10.18632/aging.205262 (PMC10756134; doi:10.18632/aging.205262)
Supplement: Supplementary Table 1 [file aging-15-205262-s002.pdf]

SUPPLEMENTARY TABLES

Supplementary Table 1. List of TLS related genes.

CETP
CCR7
SELL
CD79B
CCL19
CXCL13
LAMP3
CXCL9
CXCL11
CCL8
CCL18
CCL5
CXCL10
RBP5
CCL21
SKAP1
CD1D
CCL2
PTGDS
CCL4
CCL3
