# Supplementary material for: Prognostic role of long non-coding RNA USP30-AS1 in ovarian cancer: insights into immune cell infiltration in the tumor microenvironment
Source: Aging (Albany NY). 2023 Dec 4;15(23):13776–98. doi: 10.18632/aging.205262 (PMC10756134; doi:10.18632/aging.205262)
Supplement: Supplementary Table 3 [file aging-15-205262-s004.pdf]

Supplementary Table 3. LASSO analysis of the obtained genes and corresponding coefficients.

Variable	lambda.min
PSMB8-AS1	-0.024854969
LINC01857	-0.007698824
DTNB-AS1	-0.247195127
AC002511.2	0.036814435
TRBV11-2	-0.015803963
LINC01094	0.177261565
AP002954.1	-0.107738165
AL353699.1	0.03021951
USP30-AS1	-0.013455828
AC012236.1	-0.082114301
AL365361.1	-0.002645583
AC012181.1	0.016359778
LINC01943	-0.104857848
AL078582.1	0.212567266
MICB-DT	-0.110934382
